# Polypharmacy and potentially inappropriate medications (PIMS) in older adults referred to a liaison psychiatry service

**DOI:** 10.1192/bjo.2021.811

**Published:** 2021-06-18

**Authors:** Anietie Akpan, Omolade Longe

**Affiliations:** West London NHS Trust

## Abstract

**Aims:**

The older adult is more likely to be prescribed a lot of medications (polypharmacy) on account of multi-morbidity and being under the care of several specialists. Adverse drug events and reactions account for a significant number of acute hospital presentations in this population group with increased risks of delirium, lasting cognitive impairment, falls and death.

Medications are not routinely reviewed or rationalised in the elderly, often contributing to preventable harm.

We sought to estimate the prevalence of polypharmacy and potentially inappropriate medications, anticholinergics in particular, in patients (65 years and older) referred to the St Mary's Hospital Liaison Psychiatry Department over a 3-month period.

**Method:**

Between 01/06/2019 and 31/08/2019 all referral forms (from in-patient wards and A&E) for patients aged 65+ years were screened for medications currently prescribed and administered. The medications were confirmed via the St. Mary's Hospital electronic records, pharmacists’ completed Medicines Reconciliation and GP Summary Care Records. Polypharmacy was defined as patients prescribed 5 or more medications. Drugs with anticholinergic properties were considered as an example of Potentially Inappropriate Medication (PIMs) using the Anticholinergic Burden Scale. 77 patients were referred in the time period. 9 were excluded due to incomplete/unreconciled medication information.

**Result:**

77.94% (n = 53) were prescribed 5 or more medications.

38.24% (n = 26) were prescribed over 10 medications.

10.29% (n = 7) prescribed over 15 medications.

69% of (n = 47) prescribed an anticholinergic.

42.65% (n = 29) prescribed more than 1 anticholinergic.

**Conclusion:**

Polypharmacy and potentially inappropriate prescribing remain widespread within the older adult population.

Increased anticholinergic burden further compounds risks of cognitive impairment, delirium and death.

Other categories of Potentially Inappropriate Medications, including those no longer needed, ought to be identified and reviewed. Over-the-counter medications also need to be screened for.

Elimination or reduction of anticholinergic burden may improve quality of life for patients, as well as cost burden on services.

Pharmacovigilance, collaborative working, regular and systematic medication reviews, and on-going training are needed across services providing care for the older adult.

